# Correlates of COVID-19 Vaccine Hesitancy among a Community Sample of African Americans Living in the Southern United States

**DOI:** 10.3390/vaccines9080879

**Published:** 2021-08-08

**Authors:** Justin Xavier Moore, Keon L. Gilbert, Katie L. Lively, Christian Laurent, Rishab Chawla, Cynthia Li, Ryan Johnson, Robert Petcu, Mehul Mehra, Antron Spooner, Ravindra Kolhe, Christy J. W. Ledford

**Affiliations:** 1 Division of Epidemiology, Department of Population Health Sciences, Augusta University, Augusta, GA 30912, USA; KLIVELY@augusta.edu (K.L.L.); claurent@augusta.edu (C.L.); RCHAWLA@augusta.edu (R.C.); CYLI@augusta.edu (C.L.); RYJOHNSON3@augusta.edu (R.J.); RPETCU@augusta.edu (R.P.); mmehra@augusta.edu (M.M.); aspooner@augusta.edu (A.S.); 2 Cancer Prevention, Control, & Population Health Program, Department of Medicine, Augusta University, Augusta, GA 30912, USA; 3 Institute of Preventive and Public Health, Medical College of Georgia, Augusta University, Augusta, GA 30912, USA; 4 Department of Behavioral Science and Health Education, Saint Louis University, St. Louis, MO 63103, USA; keon.gilbert@slu.edu; 5 Department of Pathology, Section of Anatomic Pathology, Augusta University, Augusta, GA 30912, USA; rkolhe@augusta.edu; 6 Department of Family Medicine, Augusta University, Augusta, GA 30192, USA; Christy.Ledford@augusta.edu

**Keywords:** COVID-19, vaccine acceptance, race, disparities

## Abstract

In the United States, African Americans (AAs) have been disproportionately affected by COVID-19 mortality. However, AAs are more likely to be hesitant in receiving COVID-19 vaccinations when compared to non-Hispanic Whites. We examined factors associated with vaccine hesitancy among a predominant AA community sample. We performed a cross-sectional analysis on data collected from a convenience sample of 257 community-dwelling participants in the Central Savannah River Area from 5 December 2020, through 17 April 2021. Vaccine hesitancy was categorized as resistant, hesitant, and acceptant. We estimated relative odds of vaccine resistance and vaccine hesitancy using polytomous logistic regression models. Nearly one-third of the participants were either hesitant (*n* = 40, 15.6%) or resistant (*n* = 42, 16.3%) to receiving a COVID-19 vaccination. Vaccine-resistant participants were more likely to be younger and were more likely to have experienced housing insecurity due to COVID-19 when compared to both acceptant and hesitant participants, respectively. Age accounted for nearly 25% of the variation in vaccine resistance, with 21-fold increased odds (OR: 21.93, 95% CI: 8.97–5.26–91.43) of vaccine resistance in participants aged 18 to 29 compared to 50 and older adults. Housing insecurity accounted for 8% of the variation in vaccine resistance and was associated with 7-fold increased odds of vaccine resistance (AOR: 7.35, 95% CI: 1.99–27.10). In this sample, AAs under the age of 30 and those experiencing housing insecurity because of the COVID-19 pandemic were more likely to be resistant to receiving a free COVID-19 vaccination.

## 1. Introduction

The COVID-19 global pandemic has devastated many countries and, to date, is responsible for more than 144 million cases and 3 million deaths [1]. In the United States (U.S.), racial disparities in COVID-19 emerged within months of initial community spread [2,3,4,5,6,7]. African Americans (AAs) are disproportionately affected by COVID-19 mortality, comprising nearly 17.4% of total deaths while only representing 13.2% of the U.S. population [7]. Compared to non-Hispanic White Americans, AAs are nearly three times more likely to be hospitalized for COVID-19 and twice as likely to die from COVID-19 [8]. Most notably, during the early phases of the COVID-19 pandemic, communities with predominantly AA and rural populations, such as Albany, Georgia, experienced higher rates of COVID-19 hospitalizations [9,10,11,12]. Racial disparities in COVID-19 incidence, severity, and mortality have been attributed to differences in social determinants, health inequalities, and the social patterning of disease [5,11]. Coined to describe the region’s rich dark soil, the Southern Black Belt has experienced disproportionate health inequities driven by systemic racism and economic disenfranchisement [13,14,15]. Now, the Black Belt is more commonly known for clustering counties throughout the southeastern U.S. with a higher proportion of AA residents [13]. The Georgia Black Belt, which geographically ranges diagonally across the state of Georgia from the southwestern corridor (Albany area) through middle Georgia (Macon area) to central-eastern Georgia (Augusta area), has experienced disparate health outcomes over the past few decades for many diseases including, sepsis [16], infectious disease [17], cardiovascular disease [18], stroke [19], various cancers [20,21], and, more recently, COVID-19 [12,22,23]. The added burden of health inequities comes from structural features of these communities such as limited health care resources, higher area-level disadvantage, lower socioeconomic status, and a concentration of comorbidities [5,11].

### 1.1. Social Susceptibility to COVID-19

The current pandemic exposed the deep-seated inequities across many communities that show stark differences in life expectancy and risk for COVID-19. The increasing awareness of these social realities combined with a highly infectious disease indicates a syndemic problem [24,25,26]. Syndemic populations are defined by the co-occurring concentration of disease, disease interaction, and underlying social conditions [24,25,26]. For example, this syndemic problem has been observed within other infectious diseases such as HIV for AAs living in low-income southern U.S. communities [27]. Nevertheless, syndemic populations can be easily identified using a range of epidemiological tools, such as hot spotting for infectious and chronic diseases overlaid with concentrations of racial, ethnic groups across both rural and urban geographies [24,25,26]. Other epidemiological tools include the Centers for Disease Control and Prevention’s (CDC) Social Vulnerability Index [28] and the Area Deprivation Index [29], both of which identify areas such as the Georgia Black Belt as markedly vulnerable, under-resourced, and underserved communities. These tools were underused in the early stages of the pandemic, which then failed to predict the devastating impact COVID-19 would unleash on communities of color. These tools would have been instrumental in explaining the social patterning of diseases and the concentration of social determinants of health such as housing security, housing quality, food insecurity, and joblessness. The intersection of these social determinants of health hampered the ability of communities of color to adopt mitigation strategies such as physical distancing, working from home, accessing personal protective equipment (PPE), and hygiene products to keep these communities safe [30]. As a result, significant delays impeded timely public health messaging, establishing testing sites, protecting essential workers, and developing an equitable plan for vaccination. The public health impact of these structural failures reifies the salience of racism as a multilevel contributing factor to health and the lack of well-defined processes to intervene [31,32,33,34,35,36].

The growing body of evidence documenting the effects of racism on health behaviors and health outcomes helps contextualize the past 35 years of our national attention on health disparities stemming from the release of the 1985 Report of the Secretary’s Task Force on Black and Minority Health (The Heckler Report), which identified differences in Black and White health and defined this generation’s federal focus on eliminating health disparities [37]. Within this history was the discovery of the U.S. Public Health Service Syphilis Study at Tuskegee, a major bioethical failure to the core principles of public health. What has emerged from its legacy is another example of historical trauma, community harm, and the systemic disenfranchisement of Black, Indigenous, and People of Color (BIPOC) communities, which has fostered mistrust in our public health infrastructure and has woven a complex web of confusion and misinformation encumbering decisions to engage in protective behaviors to prevent and treat COVID-19 [38,39,40,41,42].

### 1.2. Health Decision-Making Susceptibility and Prevention of COVID-19

The existing health profile of BIPOC of Georgia and nationally is further complicated by a growing “infodemic” that is supported by multiple sources of misinformation that shapes public perceptions about the pandemic and the potential treatments and vaccines to mitigate its effects [43,44]. Prior to the pandemic, persistent lower rates of vaccinations existed in communities of predominantly racial and ethnic groups [45]. National polling data reports an increase in Americans expressing their intentions to receive the COVID-19 vaccine [46]. This increase in intention is up across all racial, ethnic, and gender groups. This increase across groups may be attributed to the complexity of the COVID-19 pandemic, the novelty of the disease, or systemic issues that have been observed with other vaccination efforts. In the case of COVID-19, denialism and misinformation have prompted public under-estimation of the susceptibility and severity of COVID-19 [47,48]. A survey study early in the pandemic identified reasons for COVID-19 vaccine hesitancy, including vaccine-specific concerns, need for more information, and lack of trust [49]. In the United Kingdom, COVID-19 vaccine hesitancy is associated with lower age, female gender, lower education, lower income, Black and mixed ethnicities, in a relationship or widowed, not being a homeowner, not being employed full-time, not retired, a change in working, and having a child at school [50]. COVID-19 vaccine hesitancy was also related to the perception of reduced severity of COVID-19: people who perceived a high risk of a severe COVID-19 illness course were less hesitant than people at moderate risk or low risk [50]. Conversely, when people had positive healthcare experiences, including views of healthcare workers and medicine, they were more willing to receive a COVID-19 vaccine [50].

Perceived susceptibility to COVID-19 and intention to engage in healthy behaviors are structured by knowledge, attitudes, beliefs, and the influence of important others. The risk for COVID-19 was communicated very early to affect older populations and those with chronic diseases [51,52]. Current vaccination rates are following trends of other health interventions, showing that those who view themselves as high-risk and have access to both information and nearby sites are being vaccinated [53]. Within AA communities, pre-pandemic national survey results showed AA communities had lower levels of trust and confidence in vaccines in general. Scholars who study vaccine behaviors know that previous vaccine behaviors influence current vaccine decision making. Many Americans distrust pharmaceutical companies as they view them as profit-driven, while levels of trust of the government differ by race [54]. The intersections of these complex factors set the stage for current vaccine decision making.

As of 27 February 2021, the U.S. Food and Drug Administration (FDA) authorized three vaccines for emergency use in combating the COVID-19 pandemic, including the Pfizer-BioNTech (Pfizer Inc., New York City, New York, USA & BioNTech SE, Mainz, Germany), Moderna (Moderna, Cambridge, Massachusetts, USA), and Janssen COVID-19 vaccines (Janssen Biotech Inc. of Johnson & Johnson, New Brunswick, NJ, USA) [55,56,57]. However, among the nearly 50 million U.S. persons fully vaccinated, less than 8% of those are AAs [58], despite the ongoing racial inequities in COVID-19 caseload, hospitalizations, and deaths. These trends may in part be explained by limited early access to vaccine distribution, as many of those first vaccinated were health workers. However, it is plausible that vaccine hesitancy may play a key role in the number of AAs to be vaccinated over the coming months. Studies have elucidated that AAs are three-fold more likely to be hesitant (unsure or not willing) for receiving the COVID-19 vaccine when compared with their non-Hispanic white counterparts [59]. However, to date, there is limited knowledge on the factors associated with COVID-19 vaccine hesitancy, specifically among AAs living in the southern U.S. The study aimed to examine factors associated with vaccine hesitancy among a predominantly African American community sample.

## 2. Materials and Methods

### 2.1. Study Design and Population

We performed a cross-sectional analysis of survey data collected among a subsample of participants aged 18 and older recruited within the SeroPrevalence And Respiratory Tract Assessment (SPARTA) study during community events sponsored in partnership with The 100 Black Men of Augusta. The 100 Black Men of Augusta is a local chapter of a national non-profit community organization focused on the betterment of underserved Black, Indigenous, and People of Color (BIPOC) communities. SPARTA is a multi-year prospective cohort study aimed to understand risk factors and prevalence of SARS-CoV-2 associated with COVID-19 illness and severity. Our study population included a convenience sample of 257 community members recruited during six community events in the Central Savannah River Area (CSRA): (1) 5 December 2020, in North Augusta, South Carolina; (2) 9 January 2021, in Augusta, Georgia (GA); (3) 20 March 2021, in Augusta, GA and North Augusta, South Carolina (SC); (4) 27 March 2021, in Augusta, GA; (5) 3 April 2021, in Augusta, GA and Aiken, SC; and (6) 17 April 2021, in Hephzibah, GA. This study was approved by the Institutional Review Board of Augusta University, Georgia (Protocol Number: 44818841). We obtained informed consent for all participants of the study during the community event for the collection of survey data. Study data were managed using REDCap electronic data capture tools hosted at Augusta University [60,61].

### 2.2. Primary Outcome of Interest

Vaccine hesitancy is a continuum that rests between full acceptance of all recommended vaccines and refusal of all recommended vaccines [62]. Hesitancy can indicate an individual choice to accept some and refuse some vaccines, or it can indicate the decision to delay vaccines. Lack of hesitancy should not be confused with certainty. Some people will still accept vaccines even when they are uncertain or refuse vaccines when they are uncertain [62]. With these concepts in mind, we categorized our primary variable of interest from the Likert scale survey question, “If given to you for free, how likely are you to take a COVID-19 vaccination?” Due to small sample sizes for granular Likert scale responses (i.e., very likely, likely), we defined participants into three groups: (1) acceptant (responded very likely and likely), (2) hesitant (neutral), and (3) resistant (responded unlikely and very unlikely).

### 2.3. Participant Characteristics

As a part of our anonymous 3-page participant survey, we collected data on sociodemographics, health behaviors, history of chronic health conditions, and COVID-19 beliefs and experiences. Participant demographics included age, gender, race-ethnicity, education, employment status, current health insurance status, and annual household income. Gender was determined based on multiple-choice responses from selections of “male”, “female”, and “other: open response”. Race-ethnicity was determined by self-reported response from: (a) Black or African American, (b) White, (c) Hispanic or Latino, (d) Asian, Native Hawaiian, or Pacific Islander, and (e) other (specify). Of note, because our community events were held in predominantly African American churches and communities, our sample identified as greater than 97% African American.

Health behaviors included current alcohol use, current tobacco smoking ever had a prior COVID-19 test, and ever had a flu shot. Alcohol use was determined from the dichotomous response to “In the past year, have you had at least 12 drinks of any type of alcoholic beverage? By a drink, we mean a 12 oz. beer, a 4 oz. glass of wine, or an ounce of liquor.” Current tobacco smoking was determined by yes response to the following question: “do you currently smoke cigarettes?”. History of chronic health conditions was determined by the self-reported response of “ever having a doctor” diagnosing respondents with any of the following: high blood pressure, coronary artery disease, diabetes, kidney disease, liver disease, high cholesterol, congestive heart failure, or cancer.

We identified COVID-19 beliefs and experiences based on participants’ yes/no response to the following: (1) COVID-19 knowledge—“do you believe that COVID-19 can be spread from person to person via droplets through the air?”, (2) COVID-19 safety practices —“do you wear a facemask and wash your hands regularly when in public spaces?”, (3) job loss due to COVID-19—“have you or the primary provider of your household lost a job due to the COVID-19 pandemic?”, (4) housing insecurity due to COVID-19—“have you lost your home or had difficulties paying your rent due to the COVID-19 pandemic?”, and (5) “when seeking medical care, I have encountered unfair treatment or discrimination because of my race”.

### 2.4. Statistical Analyses

We examined differences in participant characteristics comparing between vaccine hesitancy groups (i.e., resistant vs. hesitant vs. acceptant) using chi-square or Fisher Exact tests for categorical variables and Kruskal–Wallis tests for non-parametric continuous variables. To determine the association of vaccine hesitancy groups with participant characteristics, we performed multivariable polytomous logistic regression models (reference outcome: acceptant) and reported odds ratios (ORs) and associated 95% confidence intervals (CIs). The interpretations of ORs derived from polytomous logistic regression are analogous to ORs computed from binary logistic regression and can be interpreted as the log odds of having either hesitant or resistant willingness to receive a COVID-19 vaccine relative to the log odds of being acceptant (the referent outcome) per level or unit increase in a specific covariate. Further, in a polytomous logistic regression, we estimate the odds for each level of the multilevel outcome and compare these odds simultaneously with the referent outcome (e.g., acceptant). Furthermore, the polytomous model allows for analysis among all observations while controlling for associations among covariates [63]. We adjusted multivariable models for factors found significant at 0.05 alpha level from bivariate analysis, which included the following factors in the full model: age group, gender, employment health insurance, diabetes, total number of comorbidities, tobacco use, ever had a flu shot, and housing insecurity.

To estimate the total variance in COVID-19 vaccine hesitancy explained by each covariate, we calculated generalized R^2^ values using the Cox and Snell (1989) method [64]. This model follows the equation:

(1) $$R^{2}{}_{Cox\;\&\;Snell}=1-\exp\left\{\frac{-2\left[logL\left(\beta \right)-logL\left(0\right)\right]}{n}\right\}$$

where *L*(0) is the likelihood of the intercept-only model, *L*(*β*) is the likelihood of the specified model (i.e., model with covariate(s)), and *n* is the sample size. The $R^{2}{}_{Cox\;\&\;Snell}$ cannot attain a value of 1; therefore, we used the Nagelkerke (1991) adjustment to obtain “max-rescaled R^2^” values using the following formula [65]:

(2) R2Nagelkerke = 1−exp{−2[logL(β)−logL(0)]n}1−exp{−2[logL(0)]n}

We present the *R*^2^ values for both the Cox and Snell (1989) and the adjusted Nagelkerke (1991). The significance threshold was set to an alpha level of 0.05 for a two-tailed analysis. All statistical analyses were performed using SAS (version 9.4, SAS Institute Inc, Cary, NC, USA) and Stata (version 13, StataCorp LP, College Station, TX, USA).

## 3. Results

### 3.1. Characteristics of Study Population

A total of 257 participants were included in this analysis, with the majority responding as acceptant (*n* = 175, 68.1%) followed by resistant (*n* = 42, 16.3%) and hesitant (*n* = 40, 15.6%) to receiving a COVID-19 vaccination (Table 1). Vaccine-resistant participants were more likely to be younger (median age = 31 years, Interquartile range, first quartile-third quartile (IQR) = 27.0–47.0; *p*-value <0.01) when compared with acceptant (median age = 61 years, IQR = 49.0–70.0) and hesitant (median age = 46 years, IQR = 30.0–60.5) participants. COVID-19 vaccine-resistant participants were less likely to be men (45.2% vs. 57.1%, *p*-value = 0.03) when compared with acceptant participants. COVID-19 vaccine-resistant participants were more likely to be employed full-time (76.2% vs. 62.5% and 41.7% *p*-value < 0.01) and less likely to have current health insurance (61.9% vs. 82.5% and 84.0%, *p*-value < 0.01) when compared with hesitant and acceptant participants, respectively.

COVID-19 vaccine-resistant participants (median comorbidities = 0, IQR = 0–1) had lower total number of comorbidities when compared with acceptant participants (median comorbidities = 1, IQR = 0–2) (Table 2). Particularly, among resistant participants there was a lower prevalence of high blood pressure (26.2% vs. 50.9%, *p*-value < 0.01) and diabetes (4.8% vs. 21.1%, *p*-value < 0.01) when compared with acceptant participants. COVID-19 vaccine-resistant participants were more likely to be current tobacco smokers (28.6% vs. 15.0% vs. 12.0%, *p*-value < 0.01) when compared to both hesitant and acceptant participants, respectively. COVID-19 vaccine-resistant participants were less likely to had ever received a flu shot (26.2% vs. 35.0% vs. 58.3%, *p*-value < 0.01) when compared with hesitant and acceptant participants, respectively. Furthermore, resistant participants were more likely to have experienced housing insecurity due to COVID-19 (33.3% vs. 10.0% and 6.9%, *p*-value < 0.01) when compared to both hesitant and acceptant participants, respectively.

### 3.2. Likelihood of Vaccine Resistance and Hesitancy

**Figure 1:**
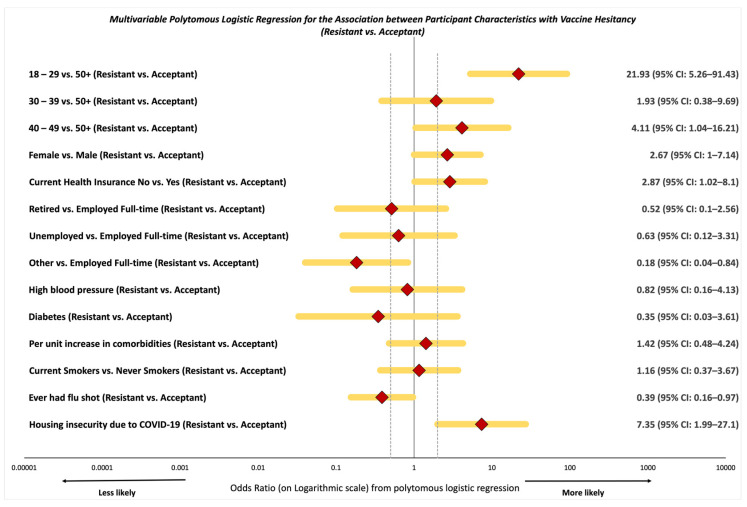
Multivariable polytomous logistic regression for the association between select participant characteristics with being vaccine-resistant vs. acceptant, among 257 community participants within the SPARTA and 100 Black Men of Augusta survey by vaccine hesitancy groups.

Participants aged 18 to 29 years had increased odds of vaccine resistance (adjusted odds ratio, AOR: 21.93, 95% CI: 5.26*–*91.43) when compared to those aged 50 and older (Figure 1 and Supplemental Table S1). Further, participants aged 40 to 49 had similar but attenuated increased odds of vaccine resistance (AOR: 4.11, 95% CI: 1.04*–*16.21) when compared to those aged 50 and older. Participants with no health insurance were nearly three-fold more likely to have vaccine resistance when compared to those with health insurance (AOR: 2.87, 95% CI: 1.02*–*8.10). Participants with other employment were less likely to be COVID-19 vaccine-resistant (AOR: 0.18, 95% CI: 0.04*–*0.84) when compared to those employed full-time. Participants that had ever received a flu shot had 61% reduced odds of vaccine resistance (AOR: 0.39, 95% CI: 0.16*–*0.97) when compared to those that had not ever received a flu shot. We observed that housing insecurity was associated with 7.3-fold increased odds of vaccine resistance (AOR: 7.35, 95% CI: 1.99*–*27.10).

**Figure 2:**
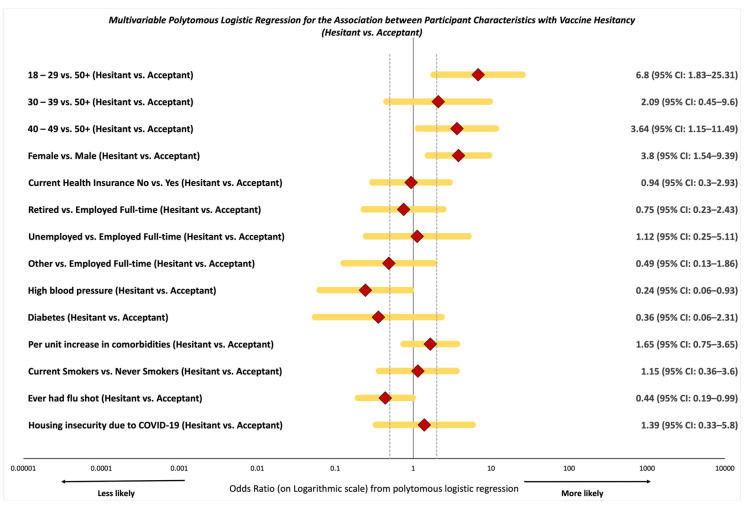
Multivariable polytomous logistic regression for the association between select participant characteristics with being vaccine-hesitant vs. acceptant, among 257 community participants within the SPARTA and 100 Black Men of Augusta survey by vaccine hesitancy groups.

We observed higher odds of vaccine hesitancy among those aged 18*–*29 years (AOR: 6.80, 95% CI: 1.83*–*25.31) when compared to those aged 50 and older (Figure 2 and Supplemental Table S1). Participants aged 40 to 49 had similar but attenuated increased odds of vaccine hesitancy (AOR: 3.64, 95% CI: 1.15*–*11.49) when compared with participants aged 50 and older. Women were more than three-fold more likely to have vaccine hesitancy when compared to men (AOR: 3.80, 95% CI: 1.54*–*9.39). Participants with a history of high blood pressure were less likely to be vaccine-hesitant (AOR: 0.24, 95% CI: 0.06*–*0.93) when compared to those with no history of high blood pressure. Those that had ever received a flu shot were less likely to be vaccine-hesitant (AOR: 0.44, 95% CI: 0.19*–*0.99) when compared to those that had not ever received a flu shot.

### 3.3. Variation in Vaccine Resistance and Hesitancy Explained by Factors

**Figure 3:**
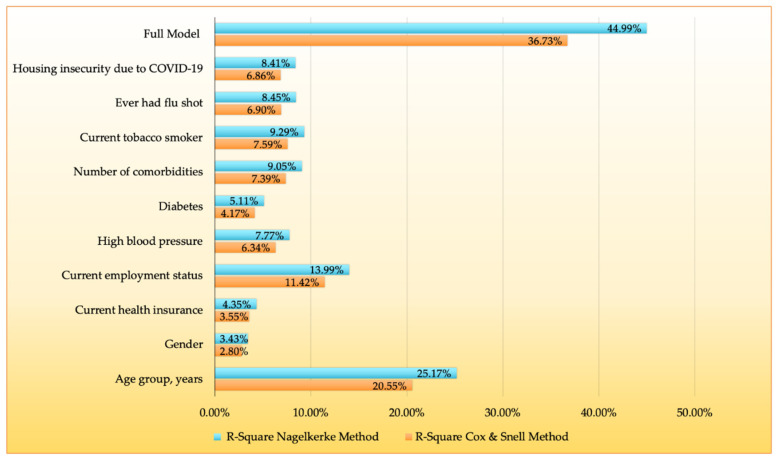
Results for R^2^ (presented as percentages) values for independently fit models and full model using the polytomous logistic regression, among 257 community participants within the SPARTA and 100 Black Men of Augusta survey by vaccine hesitancy groups.

Combined, the factors in the full model accounted for 44.99% of the variation in vaccine resistance/hesitancy (Figure 3 and Supplemental Table S2), with age groups and current employment status explaining 25% and 14%, respectively.

## 4. Discussion

In this study among mostly African Americans living in the southern U.S., we observed that nearly one-third were hesitant or resistant to receiving a free COVID-19 vaccination. Mainly, we observed that those under 30 and those experiencing housing insecurity attributed to the COVID-19 pandemic were more likely to be resistant to receiving a free COVID-19 vaccination. Overall, age was the strongest determinant of vaccine hesitancy, independently explaining nearly 25% of the variation in vaccine hesitancy. Housing insecurity was associated with greater than seven-fold increased odds of vaccine resistance and independently explained 8% of the variation in vaccine resistance. These findings highlight an important issue and necessity for innovative and proactive approaches in reaching two vulnerable populations: (1) the younger AA population that may believe that their risk of severe COVID-19 and mortality are low due to their youth and little to no chronic medical conditions, and (2) participants with housing insecurity due to COVID-19 who may have limited or no reliable interaction with health care systems.

Decision making regarding vaccination is historically linked to individual perception of the severity of the targeted disease, the consequence of not receiving a vaccination [66]. In 2011, the WHO EURO Vaccine Communications Working Group in 2011 described the “3 Cs” model, highlighting three dimensions of hesitancy: complacency, convenience, and confidence [62]. Vaccine hesitancy develops when an individual perceives a low need for a vaccination (complacency) and questions the efficacy or safety of a vaccination (low confidence) [67,68]. *Complacency* is an individual’s perception that the risks of a vaccine-preventable disease are low, which does not stimulate a need for preventive action [62]. A common case description of complacency is a mother who perceives the only risks of chickenpox (varicella) to be the discomfort associated with an itchy rash and is not familiar with the potential mortality associated with varicella [69]. This mother is vaccine-hesitant because she is not motivated to seek preventive action for a “virus of discomfort.” *Confidence* is an individual’s trust in both vaccine effectiveness and vaccine safety, trust in the system (health services and health professionals) that delivers the vaccine, and trust in the motivations of policymakers who set policy on the rules and requirements of a vaccine [62,70]. In the absence of every individual’s ability to sort through original research, critical appraise studies, and personally apply findings, individuals rely on trust relationships to make vaccine decisions. Individuals who are willing to receive a vaccine trust that the vaccine is needed, that it will work, and that it is safe [50]. The third “C,” convenience, was originally conceptualized as the ease for an individual to access a vaccine [62]. However, this is overly simplistic, especially when considering the complexities of healthcare and existing inequity. Other models further elaborate on the “5 Cs” and include *Communication,* a person’s sources for information, and *Context,* a person’s community structure, sociodemographics, and environment [71]. Further, *Constraint* is a more complex concept that describes structural and psychological barriers to vaccination, including (but not limited to) the physical availability, affordability, or geographical accessibility of the vaccine and limitations to the ability to understand (language and health literacy) vaccine information [72].

Vaccine hesitancy occurs when any of these are compromised. If an individual does not trust the sources of vaccine information, whether it is the physicians who identify a need for the vaccine for the patient, the scientists or companies who developed and tested the vaccines, or the government agencies that approved the vaccines, the patient is likely to develop vaccine hesitancy. Sources of distrust are variable, but in the context of this inquiry, lack of trust is connected to ethnicity. Among AAs, trust in the influenza vaccine and vaccine production process is lower than White American trust in the vaccine and process [73]. Confidence is especially important when a vaccine is new [74]. When people do not have personal knowledge or experience with a vaccine, they have information insufficiency and are motivated to seek additional information [75,76]. During information seeking, they may encounter disinformation, misinformation, or conspiracies, and if their confidence is low, information seekers are more susceptible to believe inaccurate information [77]. As science emerged regarding COVID-19, so did disinformation, particularly in social media, including false claims that COVID-19 is a hoax or that the virus was manufactured for the sole purpose of pharmaceutical profit [78]. Similarly, anti-vaccination sentiment spread through social media was predictive of doubt in vaccine safety [79].

Disparities in COVID-19 outcomes are explained through the complex interplay of structural inequalities, community-level protective factors, and individual decision making. Among our community sample of 257 participants, nearly half (126, 49.0%) had not received a COVID-19 screening test before participating in the COVID-19 community survey and testing event. This further speaks to how public health prevention strategies and infrastructures have largely failed to protect communities of color, low-income neighborhoods, essential workers, and those with variable employment statuses. U.S. social and health policies were slow to respond to protect vulnerable populations with extensions of unemployment benefits, providing paid sick leave, and providing clear and concise health messaging. In this study, we observed that housing insecurity due to the pandemic was significantly associated with vaccine hesitancy, thus increasing their risk for COVID-19. Communities with high concentrations of housing insecurity are also highly segregated, and many residents earn low wages and dominate essential worker categories [80,81]. For many U.S. residents living in disadvantaged neighborhoods, the COVID-19 pandemic highlighted the stark differences in livelihood when compared to those in more advantaged neighborhoods. Moreover, the COVID-19 pandemic-imposed stay-and-work-from-home orders and virtual learning throughout many communities. Those living in disadvantaged neighborhoods were faced with limitations or the inability to work remotely, isolate individual members within a household, take paid leave for COVID-19 sickness and self-quarantining, and teach young children. Local, state, federal, and various traditional and social media channels have not agreed on public health messaging to prevent the spread of COVID-19. In many cases, these inconsistencies have been plagued with misinformation, contributing to an “infodemic” and lowering the perceived susceptibility of many communities, in particular, those with lower health literacy, to understand the full scale of their risk [82,83,84]. Recent trends in the pandemic highlight that younger Americans are now highly affected. They may view their age as a protective factor because of earlier messages about risk factors for COVID-19 in its early stages. This finding aligns with the role of c*omplacency* as a factor of vaccine hesitancy—young people may perceive the risks of COVID are low, which does not stimulate a need for preventive action [62]. Perceived susceptibility influences decision making, such as testing and now vaccination.

Underlying racial inequities have undoubtedly contributed to the disproportionate COVID-19 disease burden among AAs. Such inequities are patterned by upstream structural determinants of health in the allocation of employment and housing, and it was an objective of this study to attempt to correlate those factors with vaccine hesitancy. In addition, the impact of decades of medical exploitation and abuse in the form of the Tuskegee Syphilis Study has been studied in relation to present-day mistrust of medical and research institutions [38,39]. Yet, in the context of COVID-19, it has been theorized that instances of publicized historical injustices against the AA community do not provide a full explanation for present-day hesitancy; rather, everyday racism manifesting in healthcare settings in tandem with lack of representation of AA providers are thought to be predominant contributors [85]. Thus, in addition to systems-level phenomena moderating vaccine hesitancy among AAs, future studies may also examine perceived racial discrimination as a moderating variable at the level of the clinician-patient relationship.

These findings should be viewed considering a few strengths and limitations. First, to our knowledge, this study is one of the first to examine factors related to vaccine hesitancy among a large African American community sample. Our data are based on cross-sectional survey responses, and as a result, we are unable to discern causality. Further, self-report responses are subject to misclassification. However, participants were unaware that COVID-19 vaccine hesitancy was the primary outcome of interest for this analysis, and as a result, any misclassification is likely to be non-differential by vaccine hesitancy groups. In addition, we did not examine prior COVID-19-positive test results with COVID-19 vaccine hesitancy due to limited (only nine prior positive tests) data on participants. It is likely that prior SARS-CoV2 infection may affect attitudes toward vaccination [86]. Further, there are likely many other unmeasured factors associated with vaccine hesitancy resulting in residual confounding. Nevertheless, the results of this study provide novel insight into possible factors associated with vaccine hesitancy among AAs, a population that has been disproportionately affected by COVID-19. Future studies and community programs should focus on providing resources that may attenuate the given hesitancy among younger and underserved communities.

## 5. Conclusions

In conclusion, as more of the U.S. population becomes vaccinated, vaccine resistance and hesitancy may attenuate among the young African American population attributed to increases in this population’s comfort with COVID-19 vaccine efficacy and safety. However, there are likely other contributing factors that explain this increased hesitancy, and to reduce future disparities in vaccine uptake and further COVID-19 incidence and mortality, health systems and organizations must continue to build trust and rapport with BIPOC communities through the diversity of medical professionals, community-engaged service, and being transparent about COVID-19 vaccine facts. Future studies must further examine why people who are resistant are apprehensive about receiving COVID-19 vaccinations. Lastly, we must highlight the benefits of COVID-19 vaccine immunity versus COVID-19 infection to hesitant populations.

## Figures and Tables

**Table 1:** Sociodemographic characteristics among 257 community participants within the SPARTA and 100 Black Men of Augusta survey by vaccine hesitancy groups.

Characteristic	Acceptant (*n* = 175)	Hesitant (*n* = 40)	Resistant (*n* = 42)	*p*-Value ^1^
Median age (years)—IQR ^2^	61.0 (49.0, 70.0)	46.0 (30.0, 60.5)	31.0 (27.0, 47.0)	<0.01
Age group, years—no. (%) ^3^				
18–29	10 (5.7)	10 (25.0)	18 (42.9)	<0.01
30–39	15 (8.6)	4 (10.0)	6 (14.3)	
40–49	19 (10.9)	10 (25.0)	9 (21.4)	
50+	131 (74.9)	16 (40.0)	9 (21.4)	
Male—no. (%) ^3^	100 (57.1)	14 (35.0)	19 (45.2)	0.03
Black or African American—no. (%) ^3^	168 (96.0)	38 (95.0)	42 (100.0)	0.38
Education—no. (%) ^3^				
Less than high school	15 (8.6)	1 (2.5)	2 (4.8)	0.80
High school/GED	40 (22.9)	11 (27.5)	11 (26.2)	
Some college or Associate’s degree	59 (33.7)	16 (40.0)	15 (35.7)	
College graduate	61 (34.9)	12 (30.0)	14 (33.3)	
Current employment status—no. (%) ^3^				
Employed full-time	73 (41.7)	25 (62.5)	32 (76.2)	<0.01
Retired	72 (41.1)	7 (17.5)	3 (7.1)	
Unemployed	8 (4.6)	4 (10.0)	4 (9.5)	
Other ^4^	22 (12.6)	4 (10.0)	3 (7.1)	
Current health insurance—no. (%) ^3^	147 (84.0)	33 (82.5)	26 (61.9)	<0.01
Annual household income—no. (%) ^3^				
Less than USD $20,000	28 (16.0)	12 (30.0)	7 (16.7)	0.24
USD $20,001–35,000	19 (10.9)	6 (15.0)	5 (11.9)	
USD $35,001–50,000	45 (25.7)	4 (10.0)	11 (26.2)	
USD $50,001–75,000	28 (16.0)	5 (12.5)	4 (9.5)	
USD $75,001+	29 (16.6)	5 (12.5)	4 (9.5)	
Not reported	26 (14.9)	8 (20.0)	11 (26.2)	

^1^*p*-value determined using chi-square tests, Fisher’s exact tests, and Kruskal–Wallis tests for categorical, categorical variables with small expected cell counts, and non-parametric continuous variables, respectively. ^2^ IQR denotes interquartile range. ^3^ Presented as the number and column percentage. ^4^ Other employment includes part-time, student, and disabled. ^5^ Total number of comorbidities summed from 8 baseline comorbidities.

**Table 2:** Chronic health conditions, health behaviors, beliefs, and experiences among 257 community participants within the SPARTA and 100 Black Men of Augusta survey by vaccine hesitancy groups.

Characteristic	Acceptant (*n* = 175)	Hesitant (*n* = 40)	Resistant (*n* = 42)	*p*-Value ^1^
Baseline comorbidities—no. (%) ^2^				
High blood pressure	89 (50.9)	9 (22.5)	11 (26.2)	<0.01
Chronic liver disease	2 (1.1)	0 (0.0)	0 (0.0)	0.62
Coronary artery disease	2 (1.1)	1 (2.5)	0 (0.0)	0.57
Congestive heart failure	6 (3.4)	0 (0.0)	0 (0.0)	0.24
Diabetes	37 (21.1)	3 (7.5)	2 (4.8)	<0.01
Chronic kidney disease	5 (2.9)	0 (0.0)	0 (0.0)	0.30
High Cholesterol	37 (21.1)	7 (17.5)	5 (11.9)	0.38
Cancer	9 (5.1)	1 (2.5)	0 (0.0)	0.27
Median number of comorbidities ^3^ (IQR)	1 (0, 2)	0 (0, 1)	0 (0, 1)	<0.01
Current Alcohol Use—no. (%) ^2^	70 (40.0)	18 (45.0)	25 (59.5)	0.16
Current Tobacco Smoker—no. (%) ^2^	21 (12.0)	6 (15.0)	12 (28.6)	<0.01
Ever experienced racial discrimination during medical care—no. (%) ^2^	12 (6.9)	1 (2.5)	4 (9.5)	0.43
Had prior COVID-19 test—no. (%) ^2^	92 (52.6)	19 (47.5)	20 (47.6)	0.76
Knowledge of COVID-19 spread—no. (%) ^2^	146 (83.4)	28 (70.0)	34 (81.0)	0.15
COVID-19 safety practices—no. (%) ^3^	171 (97.7)	39 (97.5)	42 (100.0)	0.61
Ever had flu shot—no. (%) ^2^	102 (58.3)	14 (35.0)	11 (26.2)	<0.01
Job loss due to COVID-19—no. (%) ^2^	23 (13.1)	3 (7.5)	9 (21.4)	0.18
Housing insecurity due to COVID-19—no. (%) ^2^	12 (6.9)	4 (10.0)	14 (33.3)	<0.01

^1^*p*-value determined using chi-square tests, Fisher’s exact tests, and Kruskal–Wallis test for categorical, categorical variables with small, expected cell counts, and non-parametric continuous variables, respectively. ^2^ Presented as the number and column percentage. ^3^ Total number of comorbidities summed from 8 baseline comorbidities.

## Data Availability

Data from this study can be made available by request from the corresponding author.

## Supplementary Materials

The following are available online at https://www.mdpi.com/article/10.3390/vaccines9080879/s1, Supplemental Table S1 Multivariable polytomous logistic regression, Supplemental Table S2 Results for R2 (presented as percentages).
