# Supplementary material for: Correlates of COVID-19 Vaccine Hesitancy among a Community Sample of African Americans Living in the Southern United States
Source: Vaccines (Basel). 2021 Aug 8;9(8):879. doi: 10.3390/vaccines9080879 (PMC8402307; doi:10.3390/vaccines9080879)
Supplement: Supplementary file 1 [file vaccines-09-00879-s001.zip › vaccines-1320698-supplementary.pdf]

Supplementary Material

Table S1. Multivariable polytomous[1] logistic regression for the association between select participant characteristics with vaccine hesitancy (referent outcome is acceptant), among 257 community participants within the SPARTA & 100 Black Men of Augusta survey by vaccine hesitancy groups.

**Hesitancy Groups**	**No. Participants**	**Acceptant (*n* = 175, 68.1%) Referent Outcome**	**Hesitant (*n* = 40, 15.6%)**		**Resistant (*n* = 42, 16.3%)**	
Variable		(%[2] or Mean, SD)	(%[2] or Mean, SD)	OR (95% CI)	(%[2] or Mean, SD)	OR (95% CI)
Age group, years						
18–29	38	26.3	26.3	6.80 (1.83–25.31)	47.4	21.93 (5.26–91.43)
30–39	25	60.0	16.0	2.09 (0.45–9.60)	24.0	1.93 (0.38–9.69)
40–49	38	50.0	26.3	3.64 (1.15–11.49)	23.7	4.11 (1.04–16.21)
50+	156	84.0	10.3	1.00 (Referent)	5.8	1.00 (Referent)
Gender						
Male	133	75.2	10.5	1.00 (Referent)	14.3	1.00 (Referent)
Female	124	60.5	21.0	3.80 (1.54–9.39)	18.6	2.67 (1.00–7.14)
Current health insurance						
Yes	206	71.4	16.0	0.94 (0.30–2.93)	12.6	2.87 (1.02–8.10)
No	51	54.9	13.7	1.00 (Referent)	31.4	1.00 (Referent)
Current employment status						
Employed full time	130	56.2	19.2	1.00 (Referent)	24.6	1.00 (Referent)
Retired	82	87.8	8.5	0.75 (0.23–2.43)	3.7	0.52 (0.10–2.56)
Unemployed	16	50.0	25.0	1.12 (0.25–5.11)	25.0	0.63 (0.12–3.31)
Other[3]	29	75.9	13.8	0.49 (0.13–1.86)	10.3	0.18 (0.04–0.84)
High blood pressure	109	81.7	8.3	0.24 (0.06–0.93)	10.1	0.82 (0.16–4.13)
Diabetes	42	88.1	7.1	0.36 (0.06–2.31)	4.8	0.35 (0.03–3.61)
Per unit increase in comorbidities[4]	-	1.07 (1.19)	0.53 (0.68)	1.65 (0.75–3.65)	0.43 (0.74)	1.42 (0.48–4.24)
Current Smokers	39	53.9	15.4	1.15 (0.36–3.60)	30.8	1.16 (0.37–3.67)
Ever had flu shot	127	80.3	11.0	0.44 (0.19–0.99)	8.7	0.39 (0.16–0.97)
Housing insecurity due to COVID-19	30	40.0	13.3	1.39 (0.33–5.80)	46.7	7.35 (1.99–27.10)

[1] The results from the polytomous logistic regression can be interpreted as the log odds of vaccine resistance or hesitance compared to the log odds of vaccine acceptance (the referent vaccine category). Model fully adjusted for age groups, gender, insurance status, employment status, high blood pressure, diabetes, comorbidities, smoking status, ever had flu shot, and housing insecurity due to COVID-19. [2] Presented as proportion within variable strata with vaccine hesitancy belief, row percentage. [3] Other employment includes unemployed, part time, student, and disabled. [4] Total number of comorbidities summed from 8 baseline comorbidities.

Table S2. Results for R2 (presented as percentages) values for independently fit models and full model using the polytomous logistic regression, among 257 community participants within the SPARTA & 100 Black Men of Augusta survey by vaccine hesitancy groups.

Variable/Model	$R^2_{Cox\ \&\ Snell}$	$R^2_{Nagelkerke}$
Age group, years	20.55%	25.17%
Gender	2.80%	3.43%
Current health insurance	3.55%	4.35%
Current employment status	11.42%	13.99%
High blood pressure	6.34%	7.77%
Diabetes	4.17%	5.11%
Number of comorbidities	7.39%	9.05%
Current tobacco smoker	7.59%	9.29%
Ever had flu shot	6.90%	8.45%
Housing insecurity due to COVID-19	6.86%	8.41%
Full Model	36.73%	44.99%

Model among 257 participants from the SPARTA & 100 Black Men of Augusta survey. Fully adjusted model contains includes age groups, gender, insured status, employment status, hypertension, diabetes, number of comorbidities, smoking status, ever had flu shot, and housing insecurity due to COVID-19. The Cox & Snell and Nagelkerke R2 values are derived using the maximum likelihood function from the fitted models of the polytomous logistic regression models. The Nagelkerke R2 is the "max rescaled" R2 which standardizes percentages to a 0–100% scale. They are analogous to the R2 values derived from the ordinary least squares regression when using linear regression. Independent R2 values do not total to 100% or to the full model R2 because there is collinearity between factors, i.e., overlap in variance explained.
